# To sleep or not to sleep? No effect of sleep on contextual word learning in younger adults

**DOI:** 10.1177/17470218231179459

**Published:** 2023-06-15

**Authors:** Emma AE Schimke, David A Copland, Sjaan R Gomersall, Anthony J Angwin

**Affiliations:** 1 School of Health and Rehabilitation Sciences, The University of Queensland, Brisbane, QLD, Australia; 2 Queensland Aphasia Research Centre, The University of Queensland, Brisbane, QLD, Australia; 3 School of Human Movement and Nutrition Sciences, The University of Queensland, Brisbane, QLD, Australia

**Keywords:** Word learning, sleep, semantic context, memory consolidation, lexical acquisition

## Abstract

This study investigated the effect of sleep on novel word learning through reading context. Seventy-four healthy young adults attended two testing sessions, with either overnight sleep (sleep group) or daytime wakefulness (wake group) occurring between the sessions. At the initial learning session, participants identified the hidden meanings of novel words embedded within sentence contexts and were subsequently tested on their recognition of the novel word meanings. A recognition test was also conducted at the delayed session. The analyses revealed comparable recognition of novel word meanings for the sleep and wake group at both the initial and the delayed session, indicating that there was no benefit of sleep compared with wakefulness for novel word learning through context. Overall, this study highlights the critical influence of encoding method on sleep-dependent learning, where not all forms of word learning appear to benefit from sleep for consolidation.

The role of sleep in memory consolidation, including some forms of new language learning, is well recognised (for reviews, see Davis & Gaskell, 2009; Rasch & Born, 2013). However, investigations into the influence of sleep on new word learning have predominantly focused on spoken word-form learning or associative learning. Given that novel words and their meaning are often acquired through context while reading (Nagy et al., 1985, 1987; Sternberg, 1987), this study investigated the influence of sleep on novel word learning via sentence context in healthy adults.

It has been established that sleeping after encoding new words can facilitate their retention or integration into the lexicon compared with an equivalent period spent awake (Dumay & Gaskell, 2007; Tamminen et al., 2010; Wang et al., 2017). Indeed, sleep benefits have been found for various forms of novel word learning, including word-form learning (Dumay & Gaskell, 2007; Tamminen et al., 2010; Wang et al., 2017), associative learning (Havas et al., 2018; Himmer et al., 2017; Mazza et al., 2016; Schimke et al., 2023; van Rijn et al., 2017), and pairing words with definitions (Kurdziel & Spencer, 2016). Such findings are often accounted for by the complementary learning systems (CLSs) model of word learning, which assumes two systems are involved in novel word acquisition and consolidation (Davis & Gaskell, 2009; McClelland et al., 1995). Rapid acquisition of a novel word is presumed to rely on hippocampal mediation, while the gradual integration of a word into the lexicon is thought to involve the neocortex. Behavioural (Dumay et al., 2004; Gaskell & Dumay, 2003) and functional magnetic resonance imaging (fMRI) studies (Davis et al., 2009; Takashima et al., 2014, 2017) provide support for the distinction between these two systems. Specifically, it has been proposed that during an offline consolidation period, the memory trace for the newly learned word undergoes repeated reactivation as it transitions from initial hippocampal acquisition to long-term integration within neocortical memory.

Although evidence largely favours a role for sleep in novel word learning (see meta-analysis by Schimke et al., 2021), there are circumstances where sleep isn’t necessary for word consolidation (e.g., Kapnoula et al., 2015; Lindsay & Gaskell, 2013; Walker et al., 2019). One factor which may influence sleep’s effect on learning is the method of encoding employed (Diekelmann et al., 2009). For example, some studies investigating implicit methods of encoding such as fast mapping or Hebb repetition learning have found no benefit of sleep on word consolidation (Himmer et al., 2017; Sobczak & Gaskell, 2019; Szmalec et al., 2012).

A recent meta-analysis identified the need for further research into sleep’s effect on different novel word encoding methods, including learning through context (Schimke et al., 2021). Contextual word learning refers to the process of deriving the meaning of an unfamiliar word from the semantic context in which it is presented. Several studies have investigated this form of word learning through silent reading in adults, finding that adults are able to acquire new words from context after limited exposures (e.g., Lowell et al., 2020; Lowell & Morris, 2017; Pagán & Nation, 2019). Contextual word learning may be considered more naturalistic than associative word learning because the meaning of the word becomes disambiguated over time. For example, in some context-based learning experiments, novel words are embedded within the final position of multiple sentences (e.g., Angwin et al., 2019; Mestres-Missé et al., 2007, 2008). In such experiments, the meaning of the word is gradually able to be inferred across sentence duplets or triplets as the sentence constraints increase (e.g., My aunt likes to read the daily dotag; A television journalist reports the dotag). Mestres-Misse et al. (2007) used event-related potentials (ERPs) to assess the acquisition of semantic information during context-based learning, and found that N400 brain potentials to the novel words were indistinguishable from real words after only three exposures within meaningful contexts. This same effect was not observed for words that were learned within contexts where no meaning could be derived.

Importantly, research suggests that contextual word learning may involve different neural correlates to associative word learning (Rodríguez-Fornells et al., 2009). Mestres-Missé et al. (2008) identified involvement of distributed brain regions including the parahippocampal gyrus, medial temporal gyrus, left anterior inferior frontal gyrus, and subcortical structures (including the thalamus and the striatum) in learning novel words from sentential contexts. Overall, as context-based learning appears to have distinct mechanisms to other encoding methods, the findings from sleep-based consolidation involving other learning paradigms cannot be assumed to generalise to contextual learning.

Research examining the impact of sleep on learning words via context is currently sparse, with much of the research focused on overnight consolidation in children (Henderson & James, 2018; Henderson et al., 2015; Henderson et al., 2021). In one study, preschool-age children learned the name of novel objects while a story book was read and then half of the children had a nap, while the other half stayed awake (Williams & Horst, 2014). It was found that sleep assisted the consolidation of the novel words and that word learning performance in the napping group even improved over time (i.e., children performed better 24 hr and 7 days later than immediately after learning the words). This same benefit was not apparent in children who did not engage in a daytime nap, suggesting a beneficial effect of sleep on novel word retention. Henderson et al. (2015) explored the impact of an overnight consolidation period on incidental word learning through a spoken story in both children and adults. Lexical competition effects were absent at immediate testing but appeared after a 24-hr consolidation period for both age groups. Furthermore, explicit recall of the novel words improved across the consolidation period. This finding is in line with other studies exploring the influence of overnight consolidation on lexical acquisition and integration. Although Henderson et al. provided important insights regarding novel word learning through context, the lack of a wake control group means the specific role of sleep cannot be elucidated.

In summary, in healthy young adults, it has been observed that sleep can benefit certain forms of word learning. However, the effect of sleep on contextual word learning in young adults remains unclear. Although there is some research demonstrating that napping enhances context-based learning of novel words in children (Williams & Horst, 2014), these findings cannot be assumed to extend to young adults due to the impact of different developmental processes. Furthermore, since contextual word learning involves different neurophysiological mechanisms to associative word learning (Rodríguez-Fornells et al., 2009), the findings from sleep-based associative learning studies cannot be generalised to this type of learning either. Thus, the aim of this study was to determine the effect of sleep on contextual word learning in healthy young adults. Healthy adults inferred the meanings of novel words embedded within sentences and were tested on their recognition of the word meanings both immediately and after a 12-hr interval containing overnight sleep or daytime wakefulness. It was hypothesised that sleep would enhance novel word recognition.

## Methods

The Human Research Ethics Committee at the University of Queensland approved the study protocol (approval number: 2018001360). All participants provided informed written consent before participating in the study.

### Participants

Healthy young adults aged between 18 and 30 years were recruited for this study. The inclusion criteria specified that participants must use English as their primary language and have normal or corrected-to-normal hearing and vision. The criteria excluded participants who reported any known or diagnosed neurological disorders, sleep disorders, mental health disorders, significant head injuries, or a history of speech, language or learning disorders. Participants were excluded if they were taking any medications with known interactions with sleep or side effects of drowsiness or medications for anxiety or depression. Participants were also excluded if they had been involved with night shift work during the past month.

This study involved undergraduate university students who participated for course credit (*n* = 79) and members of the general public (*n* = 16) who were entered into a “random prize draw” if they participated in this study. Students who were identified as not meeting the eligibility criteria (*n* = 14) were not included in the randomisation process and their data were not analysed. Data were also not analysed from participants who were later identified as not meeting the criteria (*n* = 3), participants who did not attend the delayed testing session (*n* = 3), and for not following instructions correctly for the recognition component of the contextual learning task (*n* = 1).

Sample size estimates performed with G*Power 3 (Faul et al., 2007) prior to recruitment highlighted that 29 participants per group (58 in total) would result in a power of 0.8 with *p* < .05 (based on Himmer et al., 2017 where Cohen’s *d* = 0.76). To account for potential data loss with participant attrition, we sought to recruit a total of 65 eligible participants. However, as more undergraduate students met the inclusion criteria than anticipated, seventy-four participants were included. Participants were aged between 19 and 29 years (*M* age = 21.4, *SD* = 2.7; *M* education = 15.5 years, *SD* = 1.6), and were randomly assigned to a sleep group (*n* = 39; males = 2, left-handed = 8) or wake group (*n* = 35, males = 1, left-handed = 5), as well as one of three word learning task versions. The randomisation was conducted by two independent assistants, with the use of a response-adaptive randomisation tool (Ryeznik et al., 2015) in MATLAB R2016b software (MathWorks, 2016). Randomisation was stratified based on age, sex, and number of languages used fluently.

All but one of the eligible participants were involved in a different, associative word learning study whereby those who were in the sleep group for this contextual learning study crossed over to a wake group for the associative learning study, and vice versa (Schimke et al., 2023). The word learning stimuli and paradigms used were different in each study, minimising any impact of carryover effects.

### Stimuli

#### Novel word learning task

The contextual learning paradigm was based on previous research where low and high constraint sentences duplets were used as stimuli (Angwin et al., 2019; Ripollés et al., 2014, 2017). Forty sentence pairs and 40 novel words were used as stimuli (refer to Supplementary Material for stimuli lists; Supplemental Table S1). The novel words were selected from Gupta et al. (2004) and were all two syllables in length and adhered to regular English spelling rules. The novel words were embedded into the final position of meaningful sentence duplets in the place of a real noun (e.g., Joan boiled the eggs in turos). The sentences were selected from Bloom and Fischler (1980) sentence cloze norms and were each 5 to 8 words in length. Twenty of the novel words were assigned to a “congruent” (M+) condition, where the sentence pairs led to a consistent meaning of the novel word (e.g., The earth is shaped like a karok; The children held hands and formed a karok). Such pairs were always presented in order of increasing degree of contextual constraint to ensure the meaning of the novel word was derived progressively. Specifically, the first sentence was always of low cloze probability (*M* = 28%) and the second sentence was of high cloze probability (*M* = 79%). The other 20 novel words were assigned to an “incongruent” (M–) condition, where the sentence pairs did not lead to a consistent meaning of the novel word (e.g., None of his books made any gesil; John swept the floor with a gesil). To minimise any item-specific effects for the novel word/sentence pairs, three versions of the learning and recognition tasks were created. The same novel words and sentence pairs were used across all three versions, but the items were randomly re-paired by allocating the novel words with different sentence duplets and changing the allocation of the sentences to one of the other five blocks.

#### Sleep and alertness measures

Standard sleep questionnaires were used, with the Pittsburgh Sleep Quality Index (PSQI; Buysse et al., 1989) utilised to gain information about participant sleep quality during the past month. The Morningness-Eveningness Questionnaire (MEQ; Horne & Östberg, 1976) was used to survey participant time-of-day preference. The Stanford Sleepiness Scale (SSS; Hoddes et al., 1973) and a psychomotor vigilance task (PVT; Dinges et al., 1997) were used to measure participant levels of sleepiness/alertness at each session.

Total sleep time (TST) was estimated using a device-based approach with the ActiGraph GT3X + accelerometer (ActiGraph, Pensacola, Florida). Participants were asked to wear the accelerometer on the wrist of their nondominant hand with an elastic strap, except when participating in water-based activities. Participants were required to wear device for a total of 48 hr, commencing 24 hr prior to the learning session. They continued to wear the device for 12 hr after participating in this study, as it was also worn for a separate study. A daily activity log was also kept during this time, where participants were asked to record their time in bed, time out of bed, wake time, sleep time, caffeine intake and any planned/structured exercise they participated in. ActiLife software (Version 6.13.3) was used to initialise and download data from the devices (ActiGraph, Pensacola, Florida). Devices were initialised with a 30-Hz sampling frequency, and raw acceleration data were downloaded in 60-s epochs.

#### Cognitive measures

At a separate session, participants completed a reading span task (Oswald et al., 2015) and a short version of the Attention Network Test (ANT; Fan et al., 2002). The reading span task was used to gain a measure of participant reading comprehension and complex working memory, while the ANT was used to measure the efficiency of three attention networks (alerting, orienting, and executive control).

### Procedure

#### Prior to experimental sessions

Prior to the learning session, participants were required to collect the ActiGraph device and received instructions on the device wear protocol and how to complete the daily activity log. During the wear period the participants were asked to try to sleep for at least 6-hr each night, avoid daytime napping, and try to have similar wake and sleep times each day. They were also asked to avoid drinking alcohol and taking recreational drugs.

#### Learning (Session 1)

**Figure 1.**
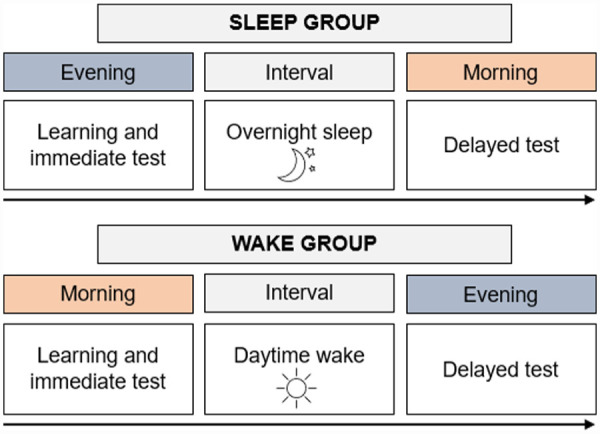
Schedule of visits.

Participants attended the learning session either in the morning (wake group) or evening (sleep group). Participants could select their preferred time of testing (i.e., either 7:00 or 8:30), to reduce circadian influences (Scullin, 2013). See Figure 1 for a depiction of the schedule of visits. Testing sessions occurred at the University of Queensland in a quiet room, with up to five people tested per session. At the beginning of the session, participants filled out the SSS and then completed the PVT via E-Prime software (Version 3.0; Psychology Software Tools, Pittsburgh, PA, 2016). The PVT consisted of a black screen, with a red dot appearing in the centre of the screen at different intervals. Participants were asked to click the left side of a computer mouse as quickly as possible every time the dot appeared on the screen. The PVT ran for approximately 10 min.

#### Contextual learning paradigm

**Figure 2.**
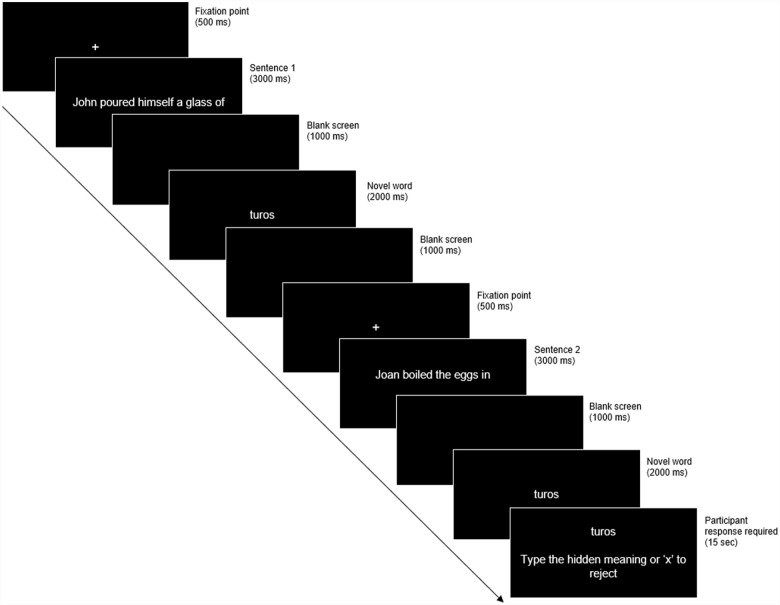
Depiction of word learning task trial.

Following the PVT, the word learning task was presented to participants via E-Prime. The task consisted of a learning phase, followed by a recognition phase. Prior to completing the word learning task, participants were provided with written and verbal instructions outlining the learning and recognition task components. Participants were asked to derive the hidden meaning of the novel words and were aware they would be tested on their recognition of the novel words following the learning phase. The learning phase involved five blocks of trials (each approx. 2–3 min in duration), with participants permitted to take a short break between the blocks. Eight sentence pairs were presented during each block and the order of sentences and block presentation was randomised for each participant. Each trial consisted of two sentences presented consecutively, followed by the same novel word (i.e., participants were exposed to the same novel word twice; Figure 2). Participants were asked to infer the meaning of the novel words that were presented with a congruent meaning (M+ condition), and to reject the meanings of the novel words that were presented with an incongruent meaning (M– condition). Each learning trial was followed by a recall screen where participants were asked to type the meaning of the novel word or reject the meaning of the word if it had no consistent meaning (by pressing the “x” key). The next trial commenced once the participant submitted their response by pressing the “enter” key or after 15s .

The learning phase was immediately followed by the recognition phase. During the recognition phase, each novel word was presented one at a time with two possible meanings under the word (one to the left and one to the right). Participants were asked to identify the meaning of the novel word as quickly and accurately as possible by selecting the correct meaning using the left and right arrow keys, or to press the spacebar to indicate that the word had no consistent meaning. Thus, participants had a one in three chance of making the correct choice. The next trial began after the participant submitted their response or after 5 s. Nonwords from the M+ condition were always presented with the correct meaning and the meaning of another nonword that had been assigned to the M+ condition within the experiment. Nonwords from the M– condition were always presented with one meaning that had been associated with the second sentence presented with that nonword during learning, and the other related to the meaning of the second sentence of a different nonword assigned to the M– condition.

Following the recognition phase, the whole task was repeated (i.e., both the learning phase and the recognition phase) to increase learning and recognition accuracy prior to the 12-hr interval, and to reduce the likelihood of participants performing below chance level at the delayed session. The same sentence pairs and novel words were presented, but the order of blocks and trials was re-randomised for each participant.

#### Delayed testing (Session 2)

The second session took place 12 hr after the first session for all participants, either in the evening (wake group) or morning (sleep group). Participants again completed the SSS and PVT at the beginning of the session. These alertness measures were followed by a test of word recognition, as per the recognition phase at the previous session. Once the recognition test was completed, participants commenced a novel word learning task for a different study. Importantly, participants had finished all aspects of the contextual word learning task reported here prior to participating in the other study.

#### Cognitive measures

Participants completed the reading span task and ANT at a separate session 12 hr after the delayed session. The reading span task was conducted via E-Prime and consisted of a letter recall component and sentence judgement component. The letter recall component involved the presentation of letters one at a time on screen and participants were asked to recall the letters in the correct order. The sentence judgement component involved the presentation of sentences on screen, and participants were required to judge whether the sentences made sense. These task components were then combined, and participants were asked to maintain sentence judgement accuracy above 85% throughout the task.

The ANT was also administered via E-Prime and involved cued reaction time (RT) and flanker components to assess the efficiency of three attentional networks: alerting, orienting, and executive control. For each trial a target arrow was presented in the centre of the screen, flanked by arrows pointing in the same (congruent trial) or opposite direction (incongruent trial). Participants were asked to identify the direction of the centre arrow as quickly and accurately as possible, by pressing the left or right computer mouse button.

### Statistical analyses

Data were analysed using SPSS (Version 27). Data skewness was within ±2 for the majority of conditions and kurtosis was within ±9, so parametric measures were used for analysis (Schmider et al., 2010). The proportion of correct responses for each learning/recognition trial and for each condition was calculated individually for each participant.

#### Coding of learning responses

Participant responses were coded as correct if they were identical to the target meaning or semantically similar to the intended target (i.e., “workplace” for “office”).E.A.E.S., A.J.A., and D.A.C. agreed on semantically similar responses that could be accepted. Spelling errors in participant responses were accepted as correct, provided they only deviated from the target word by one error (i.e., one substitution, addition, omission, or transposition). All other responses in the learning task were coded as incorrect, with five possible error types:

1. Incorrectly identifying that an M+ sentence pair had no meaning (miss).
2. Assigning a meaning to an M+ sentence pair that was incorrect or incongruent with both sentences (wrong).
3. Incorrectly assigning a meaning to an M– sentence pair (false).
4. Not providing a response (no response).
5. Other error types, for example, responses with multiple spelling errors, typing the nonword instead of the meaning of the nonword (other).

#### Accuracy at deriving the hidden meaning

For the two learning phases, a repeated-measures analysis of variance (ANOVA) was conducted for the M+ condition with Phase (1, 2) as a within-subject factor, and group (sleep, wake) as a between-group fixed factor. Accuracy for the M– condition was near ceiling after the first phase for both groups, and so no analysis was performed on the accuracy data for the M– condition.

#### Recognition testing

There were three recognition tests in total, with two conducted at the learning session and one administered at the delayed session. Recognition RTs less than 250 ms were removed, which resulted in removal of 1.18% of data for recognition 1, 0.57% of data for recognition 2, and 0.3% of data for delayed recognition. Recognition accuracy for each participant was computed based on the items the participant had identified correctly during the learning phase. Performance from Learning Phase 1 was used to calculate Recognition Test 1 accuracy and performance from Learning Phase 2 was used to compute Recognition Test 2 and Delayed Recognition accuracy (i.e., if they learned eight items correctly in Phase 1, then their Recognition Test 1 score was based on their successful recognition of those eight items only). Repeated-measures ANOVAs were conducted for the recognition trials at the initial learning session, with separate ANOVAs for each condition (M–, M+). For the delayed recognition test, independent samples *t*-tests were employed to analyse recognition accuracy between the two groups, again separately for each condition. To measure change in recognition accuracy from the initial session to the delayed session, difference scores were calculated by subtracting performance on the delayed recognition test from performance on the final recognition test (Recognition Test 2) at the learning session. Novel word RT data was also analysed using the same approach that was used for the accuracy data. Only the trials that participants had recognised accurately were included in the RT analysis.

#### Sleep and alertness measures

Independent samples *t*-tests were used to compare PSQI and MEQ scores between the two groups, as well as TST the night prior to the initial learning session. Two extreme outliers were removed from the PVT analysis for number of lapses demonstrated (one subject from each group). Separate independent *t*-tests were used for examining SSS scores and PVT lapses between groups at each session. Paired *t*-tests were also conducted to examine differences in sleepiness and lapses in attention between sessions within each group.

The data from the ActiGraph devices were analysed with ActiLife software (Version 6.13.3; ActiGraph, Pensacola, FL) using the “Batch Sleep” function, which automatically detected the sleep periods with an algorithm. Sleep period scoring was conducted with the Cole–Kripke algorithm (Cole et al., 1992) and TST was calculated for each participants’ sleep period. Data from three participants was not included in the actigraphy analysis, as they each had a sleep period which was not automatically detected. Total sleep time was compared between groups with independent *t*-tests. Daily activity logs were examined to confirm that participants did not nap (i.e., sleep during the day for more than 30 min). One participant in the wake group reporting napping between the sessions. Removal of this participant’s data did not alter the results, so their data were kept in the analysis. Pearson’s correlation analyses were conducted between recognition accuracy difference scores and TST the night between the testing sessions in the sleep group.

#### Cognitive measures

A partial reading score was calculated from each participant’s reading span task performance. Due to a recording error, data from two participants was unavailable for analysis. Data from an additional four participants was excluded from the analysis, due to performance on the sentence judgement part of the task being below the cut-off of 85%.

ANT scores for each participant were calculated using paired subtractions of the median RT from different conditions, and these represented the efficiency of the alerting, orienting, and executive control networks. The alerting score was derived by subtracting the median RT of the centre cue trials from the trials without cues. The orienting score was calculated by subtracting the median RT of the spatial cue trials from the centre cue trials. The conflict score was derived by subtracting the mean RT of the congruent trials from the incongruent trials. Higher alerting and orienting scores indicated higher alerting and orienting attention, while a higher conflict score indicated poorer executive attention. One participant was unable to attend the testing session when the cognitive tests were conducted, and thus, they did not complete the ANT and reading span task. Where there were between-group differences in any of the cognitive measures, Pearson’s correlation analyses were conducted between recognition accuracy difference scores and the cognitive measure.

## Results

### Accuracy at deriving the hidden word meanings

Both groups had comparable performance at deriving the hidden novel word meanings (Table 1). The M– condition was near ceiling for both learning trials across both groups. Overall, participants improved in their level of accuracy for the M+ condition across the two learning phases, with a main effect of phase, *F*(1, 72) = 32.74, *p* < .001, η_p_^2^ = .313, but no effect of group, *F*(1, 72) = 0.04, *p* = .843, η_p_^2^ = .001, or Group × Phase interaction, *F*(1, 72) = 0.13, *p* = .721, η_p_^2^ = .002. Error types were similar between the two groups, with the greatest percentage of the M– condition errors falling into the “false” category, and the greatest percentage of M+ errors falling into the “missing” category.

**Table 1.** Mean proportion accuracy (and standard deviations) for deriving the hidden meaning of the novel words.

Phase	M– condition		M+ condition	
	Sleep group	Wake group	Sleep group	Wake group
Learning Phase 1	.97 (.04)	.98 (.05)	.88 (.07)	.88 (.10)
Learning Phase 2	.98 (.04)	.98 (.04)	.94 (.05)	.94 (.06)

M–: incongruent meaning; M+: congruent meaning.

### Immediate recognition

**Figure 3.**
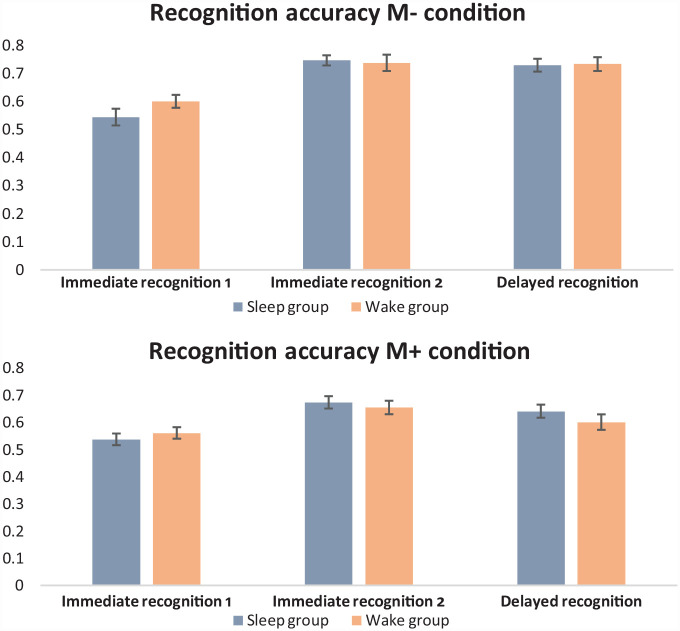
Mean proportion accuracy for the recognition task. *Note.* Error bars represent standard error of the mean (SEM).

For analysis of both the M– and M+ conditions, a main effect of test showed participants improved in their recognition accuracy from the first to the second test, M– condition, *F*(1, 72) = 62.51, *p* < .001, η_p_^2^ = .465; M+ condition, *F*(1, 72) = 38.71, *p* < .001, η_p_^2^ = .350. See Figure 3 for a depiction of the recognition accuracy data. No other main effects or interactions were evident for the analysis of either condition (Supplemental Table S2).

A similar pattern of results was also evident for the RT data (Table 2), with RTs becoming faster across tests for both the M– condition, *F*(1, 72) = 51.08, *p* < .001, η_p_^2^ = .415, and the M+ condition, *F*(1, 72) = 19.76, *p* < .001, η_p_^2^ = .215. Again, no other main effects or interactions were significant (Supplemental Table S3). These results highlight that both groups demonstrated comparable word recognition at the initial learning session, with comparable improvements in performance from the first to the second test for both the M– and M+ conditions.

**Table 2.** Mean reaction times (and standard deviations) for the recognition task.

Test	M– condition		M+ condition	
	Sleep group	Wake group	Sleep group	Wake group
Immediate recognition 1	2,748 (471)	2,813 (403)	2,506 (409)	2,559 (375)
Immediate recognition 2	2,396 (400)	2,406 (381)	2,294 (347)	2,267 (376)
Delayed recognition	2,109 (407)	2,088 (266)	1,974 (381)	2,084 (256)
Difference score	287 (318)	318 (336)	320 (325)	183 (349)

M–: incongruent meaning; M+: congruent meaning.

### Delayed recognition

To explore the effect of sleep on novel word recognition, performance at the final recognition test of the learning session (i.e., Recognition Test 2) was compared with recognition at the delayed session. For M– recognition accuracy, there was no main effect of group, *F*(1, 72) = 0.01, *p* = .940, η_p_^2^ < .001, session, *F*(1, 72) = 0.89, *p* = .348, η_p_^2^ = .012, or Group × Session interaction, *F*(1, 72) = 0.31, *p* = .581, η_p_^2^ = .004. For M+ recognition accuracy, there was a main effect of session, *F*(1, 72) = 9.38, *p* = .003, η_p_^2^ = .115, with superior recognition at the learning session, but no main effect group, *F*(1, 72) = 0.83, *p* = .365, η_p_^2^ = .011, or Group × Session interaction, *F*(1, 72) = 0.58, *p* = .448, η_p_^2^ = .008.

For M– recognition RTs, there was a main effect of session, *F*(1, 72) = 63.30, *p* < .001, η_p_^2^ = .468, with faster RTs at the delayed session, but no main effect of group, *F*(1, 72) = .004, *p* = .948, η_p_^2^ < .001, or Group × Session interaction, *F*(1, 72) = 0.17, *p* = .686, η_p_^2^ = .002. For M+ recognition RTs, there was a main effect of session, *F*(1, 72) = 41.28, *p* < .001, η_p_^2^ = .364, with faster RTs at the delayed session, but no main effect of group, *F*(1, 72) = 0.35, *p* = .557, η_p_^2^ = .005, or Group × Session interaction, *F*(1, 72) = 3.09, *p* = .083, η_p_^2^ = .041. The difference score analyses also revealed there were no significant differences between the two groups in their recognition accuracy change scores, M– condition, *t*(72) = 0.56, *p* = .581; M+ condition, *t*(72) = 0.76, *p* = .448, nor their RT change scores, M– condition, *t*(72) = 0.41, *p* = .686; M+ condition, *t*(72) = 1.76, *p* = .083.

### Sleep and alertness measures

No significant differences were found between groups for their PSQI global score or MEQ score, suggesting they were comparable with their sleep quality and time-of-day preferences. There was also no difference in TST between groups the night prior to the initial learning session. No significant correlations were found between recognition accuracy difference scores or TST between the sessions in the sleep group (M– difference score, *r* = –.001, *p* = .995; M+ difference score, *r* = –.26, *p* = .110). See Table 3 for a summary of the sleep and alertness measure scores.

**Table 3.** Summary of results for sleep-related measures.

Measure	Sleep group	Wake group	*df*	*t*	*p*
Sleep questionnaires					
PSQI (global score)	5.69 (1.91)	5.29 (1.96)	72	0.90	.370
MEQ (overall score)	52.79 (8.97)	52.77 (10.53)	72	0.01	.992
Actigraphy					
TST night between (hr)	7.22 (1.06)	–			
TST night prior (hr)	7.45 (1.18)	7.09 (0.86)	68	1.45	.152
Alertness measures					
SSS (learning session)	3.18 (1.05)	2.63 (0.77)	72	2.55	.013
SSS (delayed session)	2.36 (1.35)	2.66 (1.21)	72	1.04	.301
PVT (learning session)	2.84 (3.02)	2.24 (2.62)	70	0.91	.368
PVT (delayed session)	4.47 (4.68)	3.38 (3.77)	70	1.08	.283

PSQI: Pittsburgh Sleep Quality Index; MEQ: Morningness-Eveningness Questionnaire; TST: total sleep time; SSS: Stanford Sleepiness Scale; PVT: Psychomotor Vigilance Task.

Table displays mean values (and standard deviations) for each measure. The statistics represent the independent *t*-test results for each measure.

*t*-tests revealed a significant difference between groups in the subjective level of sleepiness at the learning session, with the sleep group reporting a greater degree of sleepiness than the wake group, *t*(72) = 2.54, *p* = .013. There was no difference in sleepiness scores between groups at their delayed session. Paired *t*-tests indicated that the sleep group reported a greater degree of sleepiness at the learning session compared with the delayed session, *t*(38) = 3.41, *p* = .002, while no difference was evident for the wake group, *t*(34) = 0.12, *p* = .909. Furthermore, while independent *t*-tests indicated no difference between groups for their PVT mean number of lapses at either session, paired *t*-tests showed both groups were more alert at the learning session compared with the delayed session, sleep group, *t*(37) = 2.20, *p* = .034; wake group, *t*(33) = 2.49, *p* = .018.

### Cognitive measures

A summary of the cognitive measure scores is provided in Table 4. Based on analysis of the partial reading score from the reading span task, the sleep group demonstrated higher working memory capacity than the wake group, *t*(65) = 2.17, *p* = .034. There were no significant correlations between the partial reading scores and M– recognition accuracy or M+ recognition accuracy difference scores in the sleep group (M– difference score, *r* = –.34, *p* = .050; M+ difference score, *r* = .10, *p* = .596) or wake group (M– difference score, *r* = –.19, *p* = .292; M+ difference score, *r* = .24, *p* = .186). Analysis of the ANT data revealed a higher orienting network score for the wake group compared with the sleep group, *t*(71) = 2.49, *p* = .015; however; there were no significant correlations between the orienting network scores and recognition accuracy difference scores in the sleep group (M– difference score, *r* = –.30, *p* = .068; M+ difference score, *r* = .05, *p* = .766) or wake group (M– difference score, *r* = .29, *p* = .087; M+ difference score, *r* = –.25, *p* = .157).

**Table 4.** Summary of results for the cognitive tests.

Task	Score	Sleep group	Wake group	*df*	*t*	*p*
Reading Span Task	Partial reading	56 (11)	50 (12)	65	2.17	.034
Attention Network Test	Alerting network	20 (21)	21 (23)	71	0.30	.766
	Orienting network	50 (24)	63 (22)	71	2.49	.015
	Conflict network	91 (29)	90 (28)	71	0.14	.889

Table displays mean values (and standard deviations) for each measure. The statistics represent the independent *t*-test results for each measure.

## Discussion

This study examined the influence of sleep on learning novel words through the semantic context of written sentences. It was predicted that recognition of the novel words at delayed testing would be enhanced by sleep compared with wakefulness; however, the results did not support this hypothesis. At the initial learning session, participants in both groups demonstrated a similar ability to derive the hidden meaning of the novel words and recognise their meanings during subsequent recognition tests. At delayed testing, after participants had either slept or stayed awake, there was also no difference in recognition accuracy between the two groups.

These results contrast with previous findings where sleep has been shown to boost novel word learning in healthy adults across a range of learning tasks (Schimke et al., 2021), including novel words associated with semantic information (Kurdziel & Spencer, 2016; Schimke et al., 2023). One explanation for this unexpected finding is that encoding new words via semantic context may not rely on sleep for consolidation. Although research has generally suggested that sleep benefits novel word learning (Schimke et al., 2021), some research has found sleep to have no influence on novel word acquisition or integration (Himmer et al., 2017; Szmalec et al., 2012). Specifically, it has been shown that novel words encoded via a fast mapping or Hebb repetition paradigm, both considered implicit learning methods, do not benefit from sleep relative to wakefulness (Himmer et al., 2017; Szmalec et al., 2012). Indeed, it has been suggested that more implicit methods of declarative learning may not rely on sleep for consolidation (Diekelmann et al., 2009).

More recently, Sobczak and Gaskell (2019) compared implicit and explicit methods of word learning and found lexicalisation was more likely to occur after an overnight consolidation period when the words were encoded via an explicit pause detection task. It is difficult to reconcile these findings with those of this study since the contextual learning paradigm wasn’t strictly an implicit encoding method. Participants were given explicit instructions to derive the hidden meanings of the novel words, and they were required to type the meanings of the novel words after each trial. Nevertheless, the nature of this task arguably involves a more ambiguous and less explicit form of learning than straight “study and learn” associative paradigms, which have been used in previous studies exploring novel word learning associated with semantics (e.g., Kurdziel & Spencer, 2016; Schimke et al., 2023). For example, while definitions were explicitly paired with novel words in Kurdziel and Spencer’s study, in this study, the participants had to infer the meaning of the novel words presented within two sentences. Moreover, as the first sentence stem was of low contextual constraint, the meaning of the novel word was not immediately obvious, and it was not until the second sentence that the word’s meaning was disambiguated.

Indeed, context-based learning is presumed to rely on distinct mechanisms to associative word learning (Mestres-Missé et al., 2007, 2008; Rodríguez-Fornells et al., 2009). Specifically, the ventral striatum appears to play an important role in this form of context-based learning (Ripollés et al., 2014, 2016; Rodríguez-Fornells et al., 2009), that is not reported for associative forms of word learning (for a review, see Tagarelli et al., 2019). Thus, it is possible that contextual learning depends on systems distinct from the CLS account for word learning and may not be as dependent on sleep for consolidation. Given this study only involved behavioural methods, we cannot identify the neurocognitive mechanisms involved or how they were affected by sleep. Neuroimaging studies exploring the influence of an overnight consolidation period on word learning have largely focused on word-form learning or associative learning (Davis & Gaskell, 2009; Takashima et al., 2014, 2017) In future, use of EEG or fMRI could possibly reveal whether there are any differences in brain activity between the two conditions after a delay that are too subtle to infer from behavioural results alone. For example, while learning was similar across the two conditions in this study, perhaps neural responses to learned words may have more closely resembled that of known words. Indeed, N400 brain potentials have been linked with novel word learning associated with semantics and through context (e.g., Angwin et al., 2014; Batterink & Neville, 2011; Mestres-Missé et al., 2007).

It is possible the lack of a beneficial effect of sleep on context-based word learning is linked to the method through which the word meanings were encoded during the learning phase. For example, in Himmer et al. (2017) and Havas et al. (2018) where sleep was found to benefit associative novel word learning, novel word-object pairings were passively encoded during the learning phase. In contract, participants in this study were required to derive the hidden meaning of the novel words during the learning phase and type the meaning at the end of each trial. Thus, it is possible that sleep may not facilitate novel word learning when participants are required to actively retrieve the meanings of the novel words during the learning phase. It should be noted that in Kurdziel and Spencer (2016) and Schimke et al. (2023) where sleep benefitted the retention of novel words associated with semantic information, participants did indeed actively type the novel words after each trial during the learning phase—however, the meaning of the word itself was not typed. Future research could directly explore differences in sleep-dependent word learning when word meanings are passive encoded versus when they are actively retrieved during the learning phase.

Research suggests that weaker/intermediate memories may benefit more from sleep than memories which have been encoded to a greater degree (Drosopoulos et al., 2007; Petzka et al., 2021; Wilhelm et al., 2012). Interestingly, although novel word recognition accuracy in this study was at a similar level to studies which have found a beneficial effect of sleep on word learning, these same benefits did not transpire in this study. For example, Himmer et al. (2017) observed a positive effect of sleep on the recognition of explicitly encoded novel word-object pairings, where mean recognition accuracy for their participants was approximately 65%–70% at the initial learning session. Similarly, in this study recognition accuracy was at 65%–67% at the learning session for the M+ condition. An interesting avenue for future research could be to manipulate pre-sleep novel word learning memory strength via a training threshold at the learning session (e.g., Denis et al., 2021; Petzka et al., 2021). Conducting such a training manipulation would enable a direct comparison between the effect of sleep on weaker versus stronger novel word encoding.

Relatedly, future research could also explore the influence of task difficulty on sleep-dependent context-based word learning. The current context-based word learning paradigm differed to that of Ripollés et al. (2014) in that while the recognition test was embedded within the Ripollés et al. learning task at the end of each learning block, in this study, the recognition test was conducted at the end of the learning phase. Accordingly, although participants within Ripollés et al. learned 40 new words (compared with 20 new words in this study), accuracy rates for the M+ condition were relatively similar between the two studies (60% in Ripollés vs. up to 67% in this study). Therefore, it is possible that learning in the current study was comparatively more difficult than in the Ripollés et al. (2014) study. Indeed, our findings were more congruent with Angwin et al. (2019), where recognition was also tested at the end of the learning paradigm and 20 words were learned (recognition rate of between 55% and 65% depending on group). It is unclear whether an easier context-based learning paradigm may be more sensitive to the effect of sleep on word learning.

To further explore the notion that context-based novel word learning may not benefit from sleep, future research could test novel word retrieval using a lexical competition paradigm. In this study, only explicit recognition of the novel words was investigated rather than the integration of the words into the lexicon. As distinct processes are involved in these testing methods, and the integration of novel words into the lexicon generally tends to emerge after sleep, it would be useful to explore this further. Although Henderson et al. (2015) showed that words learned through spoken stories rely on an overnight consolidation period for integration, it is unclear whether sleep or just the simple passing of time was responsible for this transition. Furthermore, while Williams and Horst (2014) found that pre-schoolers’ novel word acquisition from stories was boosted by napping (Williams and Horst, 2014), there were several key differences to this study (e.g., population, sleep type, stimuli, and procedure used) which make it difficult to draw inferences.

In this study, associations between novel word recognition and sleep duration, attentional capacity, and working memory ability were also explored. There were no associations between recognition performance difference scores and TST the night between the sessions in the sleep group. Although participants in the sleep group demonstrated higher working memory capacity than participants in the wake group, no correlations were found between working memory and recognition accuracy in either group. Regarding attentional capacity, the participants in the wake group showed higher orienting attention than the sleep group, but again there were no correlations found between the orienting score and recognition performance.

As the sleep and wake groups were tested at different times of the day, we cannot dismiss the influence of time-of-day effects on the present findings. It is understood that circadian influences can potentially affect encoding and/or retrieval (Schmidt et al., 2007). There was a difference in self-reported levels of sleepiness between groups at the encoding session, with the sleep group reportedly sleepier than the wake group. However, the PVT results did not corroborate these findings. Nonetheless, the impact of circadian effects could be controlled for in future with the use of a nap design.

This study has implications for language learning through the context of naturalistic reading. First, the results highlight that adults can learn new vocabulary from context after relatively few exposures and retain the vocabulary even after a 12-hr delay. The findings also contribute to a clearer understanding of the encoding conditions under which sleep appears to influence language acquisition. It appears that explicit recognition of novel words learned through semantic context does not rely on a sleep period for overnight consolidation and that similar reductions in retention of novel words is seen through the simple passing of time, regardless of whether wake or sleep follows. Finding out under which conditions sleep-based novel word learning operates can help with timing of vocabulary acquisition and potentially has implications for therapeutic intervention, where some types of language intervention may be timed depending on sleep benefits. As this is a relatively underexplored area and this study was the first to directly explore the influence of sleep on learning new words through context, the results highlight the need for future research in this area.

## Conclusion

This study was the first to explore the influence of sleep on context-based novel word learning in healthy adults, with a wake group for comparison. Overall, there were no differences between groups in their recognition of the novel words, regardless of whether the interval between the sessions was filled with sleep or wakefulness. These findings suggest that context-based novel word learning may not preferentially rely on sleep for consolidation. Further research is required to elucidate the mechanisms underpinning this process, as well as whether sleep is required for the lexical integration of words learned through context.

## Supplemental Material

sj-docx-1-qjp-10.1177_17470218231179459 – Supplemental material for To sleep or not to sleep? No effect of sleep on contextual word learning in younger adults Supplemental material, sj-docx-1-qjp-10.1177_17470218231179459 for To sleep or not to sleep? No effect of sleep on contextual word learning in younger adults by Emma AE Schimke, David A Copland, Sjaan R Gomersall and Anthony J Angwin in Quarterly Journal of Experimental Psychology
