# Supplementary material for: To sleep or not to sleep? No effect of sleep on contextual word learning in younger adults
Source: Q J Exp Psychol (Hove). 2023 Jun 15;77(4):789–802. doi: 10.1177/17470218231179459 (PMC10960315; doi:10.1177/17470218231179459)
Supplement: sj-docx-1-qjp-10.1177_17470218231179459 – Supplemental material for To sleep or not to sleep? No effect of sleep on contextual word learning in younger adults [file sj-docx-1-qjp-10.1177_17470218231179459.docx]

Supplementary Material for:

**To Sleep or Not to Sleep? No Effect of Sleep on Contextual Word Learning in Younger Adults**

Emma A. E. Schimke^1^, David A. Copland^1,2^, Sjaan R. Gomersall^1,3^, and Anthony J. Angwin^1^

^1^School of Health and Rehabilitation Sciences, The University of Queensland, Brisbane, QLD, Australia

^2^Queensland Aphasia Research Centre, The University of Queensland, Brisbane, QLD, Australia

^3^School of Human Movement and Nutrition Sciences, The University of Queensland, Brisbane, QLD, Australia

**Author Note**

Emma A. E. Schimke
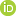
 https://orcid.org/0000-0002-3647-9450

David A. Copland
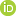
 https://orcid.org/0000-0002-2257-4270

Sjaan R. Gomersall
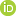
 https://orcid.org/0000-0001-6808-0180

Anthony J. Angwin
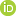
 https://orcid.org/0000-0003-4547-9433

Correspondence concerning this article should be addressed to Emma Schimke, School of Health and Rehabilitation Sciences, The University of Queensland, Brisbane, QLD, Australia, 4072. Email: [e.schimke@uq.edu.au](mailto:e.schimke@uq.edu.au)

**Table S1**

*Stimuli List for the Contextual Word Learning Task (Version 1)*

| **Condition** | **Sentence 1** | **Sentence 2** | **Novel word** | **Meaning** |
| --- | --- | --- | --- | --- |
| **Block 1** | | | | |
| M+ | Vanessa studied to become a | Perry was sick so he visited the | terid | doctor |
| M+ | When the war ended there was | A dove is a symbol of | tefob | peace |
| M+ | Sometimes success is simply a matter of | The gambler had a streak of bad | digis | luck |
| M+ | Some of the ashes dropped on the | Ants mostly live under the | debel | ground |
| M– | The crime rate has gone up this | He shouted at the top of his | kegal | lungs |
| M– | On his vacation he got some needed | At night the old woman locked the | gigin | door |
| M– | During the volley Joe twisted his | The boat passed easily under the | kudil | bridge |
| M– | They rested under a tree in the | The lawyer gave Sally his business | togot | card |
| **Block 2** | | | | |
| M+ | The Smiths had never visited that | New York is a very busy | posek | city |
| M+ | There are many cultures on | Animals live on the planet | gabek | earth |
| M+ | John poured himself a glass of | Joan boiled the eggs in | turos | water |
| M+ | They took short trips during the | Catherine jogged during her lunch | bonan | break |
| M– | Joan fed her baby some warm | Gold is a very precious type of | batus | metal |
| M– | Plants will not grow in dry | The hot sand burnt his | padod | feet |
| M– | Her new shoes were the wrong | Yesterday they canoed down the | tusen | river |
| M– | Coming in he took off his | The captain said to drop the | kumit | anchor |
| **Block 3** |  |  |  |  |
| M+ | John needed a battery for his | On his left wrist he wore a | bekor | watch |
| M+ | My aunt likes to read the daily | A television journalist reports the | tasol | news |
| M+ | Dick waited to read a | Dick wrote a chapter in the | binis | book |
| M+ | Jill looked back through the open | Tim threw a rock and broke a | dotag | window |
| M– | Don found that he had no spare | Ray fell down and skinned his | gesil | knee |
| M– | The kids fed the ducks some stale | The wealthy child attended a private | pavat | school |
| M– | The pill contained a powerful | They raised pigs on their | bokas | farm |
| M– | Peter could not close his | Father carved the turkey with a | tosik | knife |
| **Block 4** |  |  |  |  |
| M+ | Phill gets headaches from drinking | The new cafe makes really good | balen | coffee |
| M+ | She dropped a glass and scared the | The mother gave birth to a new | denet | baby |
| M+ | Few nations are now ruled by a | In the palace lives the king and | dekop | queen |
| M+ | Cheryl burned the roof of her | Jimmy blew cigarette smoke from his | pibet | mouth |
| M– | The cigar burned a hole in the | She bought milk from the corner | garol | store |
| M– | None of his books made any | John swept the floor with a | tinuf | broom |
| M– | The old house was built entirely of | Motorcycles can create a lot of | dekim | noise |
| M– | He had to fill his truck with | He mailed the letter without a | tutin | stamp |
| **Block 5** |  |  |  |  |
| M+ | He disliked having to commute to the | She called her husband at his | dalid | office |
| M+ | Most students prefer to work during the | Most cats see very well at | karok | night |
| M+ | The earth is shaped like a | The children held hands and formed a | keseg | circle |
| M+ | The apple pie had a delicious | A dog has a good sense of | kirop | smell |
| M– | The lady at the church played the | The pigs wallowed in the | koseg | mud |
| M– | While skiing Randy broke his | The man was caught selling an illegal | tonap | drug |
| M– | Every month Rick had to clean his | A cruise ship crossed the | kelib | ocean |
| M– | Our new green car blocked the narrow | The winter was very harsh this | dunas | year |

*Note.* M– = congruent meaning, M+ = incongruent meaning. Meaning for the M– condition represents the meaning of the second sentence. Meaning for the M+ condition represents the meaning of both sentences.

**Table S2**

*Repeated-Measures ANOVA Results for Recognition Accuracy at Initial Learning Session*

| Condition | Variables | *F*(1,72) | *p* | *η_p_^2^* |
| --- | --- | --- | --- | --- |
| M– | Group | 0.67 | .415 | .009 |
|  | Test | 62.51 | < .001 | .465 |
|  | Group x Test | 2.27 | .136 | .031 |
| M+ | Group | 0.01 | .928 | < .001 |
|  | Test | 38.71 | < .001 | .350 |
|  | Group x Test | 1.32 | .254 | .018 |

*Note.* ANOVA = analysis of variance, M– = incongruent meaning, M+ = congruent meaning.

**Table S3**

*Repeated-Measures ANOVA Results for Recognition Response Times at Initial Learning Session*

| Condition | Variables | *F*(1,72) | *p* | *η_p_^2^* |
| --- | --- | --- | --- | --- |
| M– | Group | 0.22 | .643 | .003 |
|  | Test | 51.08 | < .001 | .415 |
|  | Group x Test | 0.27 | .607 | .004 |
| M+ | Group | 0.04 | .844 | .001 |
|  | Test | 19.78 | < .001 | .215 |
|  | Group x Test | 0.51 | .476 | .007 |

*Note.* ANOVA = analysis of variance, M– = incongruent meaning, M+ = congruent meaning
